# A patient with a long history of relapsing psychosis and mania presenting with anti-NMDA receptor encephalitis ten years after first episode

**DOI:** 10.1590/1980-57642015DN93000016

**Published:** 2015

**Authors:** Mateus Mistieri Simabukuro, Christian Henrique de Andrade Freitas, Luiz Henrique Martins Castro

**Affiliations:** 1 Neurology Division, Hospital das Clínicas da Faculdade de Medicina da Universidade de São Paulo, São Paulo SP, Brazil.

**Keywords:** anti-NMDA receptor encephalitis, psychosis, bipolar affective disorder, relapses

## Abstract

Anti-N-methyl-*D*-aspartate receptor (NMDAR) encephalitis is a recently discovered autoimmune disorder, in which antibodies target NMDARs in the brain, leading to their removal from synapses. Early in the disease course, patients often present with marked psychosis and mood disturbances (i.e. mania, depression), explaining why most of these patients are first seen by psychiatrists. Hence, autoimmune encephalitis is receiving growing attention from psychiatry, mainly owing to concerns over misdiagnosing immunomediated and potentially curable disorders as primary psychiatric disorders, such as schizophrenia or major depressive disorder. Although anti-NMDAR encephalitis occurs in the context of new-onset psychiatric symptoms, there is a lack of information on differential diagnosis and treatment of this disorder after a long-term diagnostic history of functional psychiatric disorders. We report a case of a patient with a long history of bipolar affective disorder evolving with anti-NMDAR encephalitis, initially misdiagnosed as non-organic psychosis.

## Abstract (pt)

A encefalite antirreceptor N-metil-D-aspartato (NMDA) é uma doença autoimmune recentemente descoberta, na qual anticorpos têm como alvo os receptors NMDA no cérebro, levando à sua remoção da sinapse. Os pacientes frequentemente desenvolvem psicose e distúrbios de humor proeminentes nas fases iniciais da doença (i.e: mania e depressão), o que explica o fato de que a maioria destes pacientes seja vista primeiramente por psiquiatras. Consequentemente, as encefalites autoimunes têm recebido atenção crescente pela psiquiatria, principalmente pela preocupação de erroneamente diagnosticar doenças imunomediadas e potencialmente tratáveis como doenças psiquiátricas primárias, como esquizofrenia ou transtorno depressivo maior. Embora a encefalite antirreceptor NMDA ocorra no contexto de sintomas psiquiátricos de início recente, há carência de informações sobre o diagnóstico diferencial e tratamento desta doença após o diagnóstico de doenças psiquiátricas funcionais de longa data. Aqui, nós apresentamos o caso de uma paciente com diagnóstico de transtorno afetivo bipolar de longa data evoluindo com encefalite antirreceptor NMDA, inicialmente diagnosticada erroneamente como uma psicose não-orgânica.

## CASE REPORT

A 34-year-old, right-handed woman was transferred to the Emergency Department of the Institution from a psychiatric ward for investigation of Fever of Unknown Origin (FUO). She had a past medical history of bipolar affective disorder and was recently admitted to a psychiatric ward after a worsening of psychiatric symptoms in the preceding weeks.

Ten years earlier, a few months after having given birth to her first child, she developed behavioral changes and symptoms of language dysfunction, characterized by euphoria evolving with depressive symptoms and writing difficulties. She became more distant from her baby albeit without negative feelings towards him. There were episodes suggestive of auditory hallucinations: she woke up in the middle of the night, claiming she had heard people talking loud in the living room. The patient evolved with an isolated episode of unwitnessed seizure, and underwent investigation with brain Magnetic Resonance Image (MRI), cerebrospinal fluid (CSF) examination and electroencephalogram (EEG), which disclosed no remarkable findings. Treatment with carbamazepine was started and she improved in the ensuing 8 months, resuming all previous activities.

Seven years before admission she stopped carbamazepine when realizing she was pregnant. Pregnancy and delivery occurred with no complications.

Psychiatric symptoms relapsed 4 years before admission, having developed persecutory delusions (she feared leaving the house, thinking someone was trying to kill her; episodes of dangerous driving by speeding because she thought she was being pursued), hyper-religiosity (praying and reading the Bible constantly, although she never had been a very religious person) and insomnia. At this time she was seen by a psychiatrist and received the diagnosis of bipolar affective disorder and was started on valproate leading to improvement in symptoms in the months that followed.

The patient had been well until 6 months before, when she started complaining of fatigue and insomnia, forcing her to give up her job as a journalist.

Forty-five days before admission, she noticed diminished taste and smell sensations. In the ensuing days she evolved with behavioral changes, visual and auditory hallucinations and periods of aggressive acting out without known precipitating factors. Her husband described episodes in which she woke up in the middle of the night, agitated, insisting she was leaving to appear in a TV show and reported episodes where she could not sleep because she believed there were people having conversations in the living room.

She began having meals at unusual hours, repeating the up to 3 times within a 1-2 hour period; sometimes unable to remember having already eaten.

Her husband also noticed frequent soliloquy and an episode when she saw an imaginary bed sheet floating above her head. Sometimes she made incomprehensible and meaningless statements. The patient was reevaluated by her psychiatrist who prescribed valproate, quetiapine, venlafaxine, alprazolam; however symptoms worsened.

She began to write a diary to record her symptoms because she noticed her memory was affected. Psychiatric symptoms culminated in an event in which she saw her deceased mother through the window and invited the housekeeper to jump with her from the 10^th^ floor. Shortly afterwards, she was admitted to a psychiatric ward where treatment with aripiprazole, lithium and haloperidol (unknown doses) was started.

Ten days later, she presented with fever and tachycardia and was transferred to our Institution. No history of social drinking, use of tobacco and other recreational drugs was reported. There were no cases of psychiatric or neurologic diseases in her family.

At admission her pulse was 78 beats per minute, blood pressure 110/72 mm Hg, and axillar temperature was 39ºC. On neurologic examination she had open eyes, exhibited psychomotor agitation (with need for mechanical restraints), had incoherent speech, and could not follow commands. There were neither signs of hypertonia nor of meningeal irritation. The remainder of the physical and neurological examination was unremarkable.

Results of a complete blood count and white cell differential count were normal, and serum levels of creatinine was 1.44 mg/d, Urea:58 mg/dL, Sodium: 155 mEq/L, Potassium 4.4 mg/dL, C-Reactive protein 8.3 mg/d. Erythrocyte Sedimentation Rate: 2mm, creatine phosphokinase 359 U/L, serum lithium level was normal. Serology for HIV and syphilis were negative, thyroid function was normal as were results of tests for rheumatic diseases. A brain MRI disclosed no remarkable findings and lumbar puncture was traumatic showing 2400 red blood cells per mm^3^, 14 white cells per mm^3^ (79% neutrophils, 13% lymphocytes, 6% monocytes, 1% eosinophils, 1% basophils), while glucose and protein levels were normal. An EEG disclosed diffuse disorganization, irregular theta and delta slow waves that sometimes occurred in bursts, predominantly in right frontotemporal regions, with no epileptiform activity recorded.

Eight days after admission, the patient presented with orofacial dyskinesias, and a working diagnosis of anti-NMDAR encephalitis was reached. In the days that followed she evolved with pneumonia, respiratory insufficiency, needing orotracheal intubation and was admitted to the intensive care unit (ICU). Generalized seizures and intense dysautonomia ensued.

Investigation for ovarian teratoma and other tumors with computed tomography (CT) of the thorax, abdomen and pelvis was performed was negative.

Immunotherapy with plasmapheresis (6 sessions) was started empirically on alternating days, before return of neuronal antibody results.

On the day of the last plasmapheresis session, the results were received for NMDAR antibodies, testing positive in both serum and CSF. There was gradual improvement following treatment: after twelve days she could obey commands and write her name. Results of the follow-up bedside cognitive evaluation with the Mini-Mental State Examination and Brief Battery are summarized in Table 1. The patient had a 49-day ICU stay and developed several complications (sepsis, phenytoin skin reaction, sacral pressure ulcer). Discharge occurred 66 days after admission and the patient continued improving.

**Table 1:** MMSE and Brief Cognitive Battery evolution during follow-up.

Follow-up duration (in days)		D49	D56	D167
Mini-Mental State Examination		19	25	**29**
Brief Cognitive Battery	Incidental Memory	8	8	**8**
	Immediate Recall	4	7	**9**
	Learning	6	6	**10**
	Delayed Recall	5	8	**10**
Verbal Fluency	Semantic (animals)	8	12	**14**
	Phonemic (letter ”p”)	8	11	**18**

At the last visit (12 months of follow-up) a remarkable recovery was evident: she is able to drive and take care of her children, but did not go back to work. No further immunotherapy or chronic immunosuppression was needed, and she is on valproate alone.

## DISCUSSION

To our knowledge, this is the second comprehensive description of a patient with long-term diagnosis of mania and multiple relapses of psychiatric symptoms, in which the last episode was diagnosed as anti-NMDAR encephalitis.^1^ This case is important for several reasons. Firstly, diagnosis was delayed because neuropsychiatric symptoms were attributed to a previously existing, long-standing psychiatric disorder. Since its description, anti-NMDAR encephalitis has attracted great interest from the psychiatric community because the marked psychosis early in the disease's multistage course raises the possibility of an identifiable, organic and treatable subgroup of psychosis.^2^ Patients rarely have isolated psychiatric presentation where, one month after disease onset, most of them have four or more of the following group of 8 symptoms: behavior and cognition, memory, speech, seizures, movement disorder, loss of consciousness, autonomic dysfunction and central hypoventilation.^3^

In the present case, there were subtle but clear early signs of neurologic dysfunction:

[1] she wrote a diary of her complaints because she was aware that her memory was impaired;
[2] after hallucinations, she developed periods of a catatonia-like state, which is a remarkable finding in NMDAR encephalitis;
[3] autonomic dysfunction (i.e. hyperthermia and tachycardia) was initially misattributed to a neuroleptic malignant syndrome. Unfortunately, the occurrence of alterations in CSF and on EEG were overlooked: they were initially misattributed to traumatic lumbar puncture (CSF) and to medications and septic encephalopathy (EEG). It was only after orofacial dyskinesias appeared that anti-NMDAR encephalitis was considered as a differential diagnosis, resulting in treatment delay. Even subtle abnormalities in CSF (mild pleocytosis, slightly increased protein concentration) and inspecific EEG abnormalities should be interpreted as "red flags" indicating an organic cause in patients with psychiatric symptoms.^4^ Therefore, the importance of familiarity with this syndrome among psychiatrists should be emphasized, because recognition of the combinations of these symptoms should raise suspicions of anti-NMDAR encephalitis and prompt testing for antibodies against the GluN1 subunit of the NMDAR.

Second, an intriguing question is whether the previous psychiatric episodes were in fact associated with anti-NMDAR encephalitis. To address this possibility, two questions must be answered:

[1] Can patients with anti-NMDAR encephalitis improve spontaneously? and
[2] Can the disease relapse? In fact, although immunotherapy is deemed a cornerstone for the treatment of autoimmune encephalitis,^5^ there are reported cases of recovery without specific treatment.^3,6,7^ In a cohort of 501 patients, 29 were not treated with immunotherapy or surgery. The reasons for this absence of treatment were that the disease was diagnosed during relapses in half of these patients (initial episodes untreated); diagnosis was retrospective in 9 patients (four post-mortem and five with stored serum or CSF samples); and spontaneous improvement occurred within a few weeks in 6 patients. This cohort also showed that 45 patients had clinical relapses during a follow-up of 24 months (a 12% risk), and among those who relapsed, 33% had multiple relapses. Most of the relapses were less severe, more often mono-symptomatic, and resulted in fewer admissions to the ICU in comparison to the initial episode.^3^ Moreover, our patient presented with language dysfunction and a putative episode of seizure during the first psychiatric episode, inconsistent with a non-organic cause. However, because CSF or serum samples from previous episodes were unavailable to perform a retrospective NMDA antibody analysis, this question will remain unanswered.

Anti-NMDAR encephalitis is increasingly being recognized earlier in patients with new-onset psychiatric episodes. The present case illustrates that autoimmune encephalitis should not be overlooked during evaluation of patients with long-term course of psychiatric disease, because even these patients could harbor neuronal antibodies and therefore have a potentially curable and reversible disease.
